# Acetylcholine-Binding Protein in the Hemolymph of the Planorbid Snail *Biomphalaria glabrata* Is a Pentagonal Dodecahedron (60 Subunits)

**DOI:** 10.1371/journal.pone.0043685

**Published:** 2012-08-20

**Authors:** Michael Saur, Vanessa Moeller, Katharina Kapetanopoulos, Sandra Braukmann, Wolfgang Gebauer, Stefan Tenzer, Jürgen Markl

**Affiliations:** 1 Institute of Zoology, Johannes Gutenberg University, Mainz, Germany; 2 Institute of Immunology, University Medical Center of the Johannes Gutenberg University, Mainz, Germany; Russian Academy of Sciences, Institute for Biological Instrumentation, Russian Federation

## Abstract

Nicotinic acetylcholine receptors (nAChR) play important neurophysiological roles and are of considerable medical relevance. They have been studied extensively, greatly facilitated by the gastropod acetylcholine-binding proteins (AChBP) which represent soluble structural and functional homologues of the ligand-binding domain of nAChR. All these proteins are ring-like pentamers. Here we report that AChBP exists in the hemolymph of the planorbid snail *Biomphalaria glabrata* (vector of the schistosomiasis parasite) as a regular pentagonal dodecahedron, 22 nm in diameter (12 pentamers, 60 active sites). We sequenced and recombinantly expressed two ∼25 kDa polypeptides (*Bg*AChBP1 and *Bg*AChBP2) with a specific active site, N-glycan site and disulfide bridge variation. We also provide the exon/intron structures. Recombinant *Bg*AChBP1 formed pentamers and dodecahedra, recombinant *Bg*AChBP2 formed pentamers and probably disulfide-bridged di-pentamers, but not dodecahedra. Three-dimensional electron cryo-microscopy (3D-EM) yielded a 3D reconstruction of the dodecahedron with a resolution of 6 Å. Homology models of the pentamers docked to the 6 Å structure revealed opportunities for chemical bonding at the inter-pentamer interfaces. Definition of the ligand-binding pocket and the gating C-loop in the 6 Å structure suggests that 3D-EM might lead to the identification of functional states in the *Bg*AChBP dodecahedron.

## Introduction

Acetylcholine-binding proteins (AChBP) have been described in several gastropods [1]–[3] and, more recently, also in an annelid [4]. They are homo-pentamers and water soluble homologues of the extracellular, pentameric ligand-binding domain (LBD) of the membrane-bound nicotinic acetylcholine receptors (nAChR) that belong to the Cys-loop receptor superfamily [5]–[7]. The AChBP subunit is homologous to the α7-subunit of nAChR-LBD [1], [8]. In AChBP, the pentameric quaternary structure and the overall subunit features of nAChR-LBD are fully conserved; this also includes the typical pocket-like active site at the five inter-subunit interfaces, with several strictly conserved aromatic residues for ligand binding, and a C-loop for controlled access to the active site. The pocket interface typically shows a “principal side” with highly conserved residues and a “complementary side” with greater residue variability [3]. This provides multiple means for selective binding of agonists and antagonists, including some powerful toxins such as conotoxin, bungarotoxin, cobratoxin and strychnine. Consequently, due to their experimental advantage as soluble proteins and being structural and functional surrogates of the much less accessible nAChR-LBD, a wealth of agonist- and antagonist-binding data is available for AChBP, and a remarkable panel of corresponding crystal structures has been deposited in the databases. This has greatly stimulated the entire nAChR research field [9]–[11].

With respect to the biological function of AChBP in molluscs, a possible role as suppressor of cholinergic transmission has been proposed. AChBP molecules are expressed in, and secreted from, glial cells and appear to be released into the synaptic cleft of cholinergic neurons where they are able to block synaptic transmission in cell cultures [8]. This observation has been challenged by others, who found that *in vivo* the AChBP producing glial cells are localized in the vicinity of neuronal cell bodies rather than close to synapses; they proposed that AChBP might instead regulate non-synaptic transmission [12]. A variant of AChBP, the di-pentameric amorphous calcium carbonate-binding protein (ACCBP) detected in some molluscs, is involved in the regulation of shell and nacre growth [13]. Results from the pearl oyster *Pinctada furcata* and the abalone *Haliotis discus hannai* indicate that ACCBP recognizes different CaCO_3_ crystal phases and prevents random calcium carbonate crystallization in the supersaturated hemolymph and extrapallial fluid. Moreover, it is likely to control the typical growth patterns of the molluscan shell [13], [14]. Binding to amorphous CaCO_3_ is presumably achieved *via* the glycan-rich moiety typical for ACCBP, and somehow triggered by conformational changes caused by ligand (acetylcholine) binding [13]. A further biologically relevant property of AChBP is the ability to bind algal phytotoxins (such as spirolides and gymnodimines) in their active site pocket at picomolar affinities [15]. Therefore, AChBP might serve for protection against phytotoxins in the hemolymph and, *vice versa*, such phytotoxins might represent broad-range AChBP and nAChR antagonists to repel algae feeders.

*Biomphalaria glabrata* is a tropical freshwater gastropod of the planorbid family. It has been intensively studied due to its crucial role as intermediate host of *Schistosoma mansoni*, the trematode parasite causing the severe tropical disease bilharziosis. Previously, our research group published the structure of multimeric *B. glabrata* hemoglobin and described a second hemolymph protein of similar size (20–25 nm), but of rosette-like appearance [16]. This “rosette protein” had previously been observed in the planorbid genera *Planorbis*, *Planorbarius*, *Planorbella* and *Helisoma*, but was mistaken by these authors for hemoglobin [17]–[21]. Our previous study revealed that it is immunologically and spectroscopically distinct from *B. glabrata* hemoglobin and contains a 31 kDa major and a 25 kDa minor polypeptide subunit [16].

In the present study, mass spectrometry of the 31 kDa polypeptide suggested a relationship to AChBP, and thereby we identified two distinct *Bg*AChBP subunits that we termed *Bg*AChBP1 and *Bg*AChBP2. By DNA sequencing and phylogenetic tree analysis, we traced the evolution of the two subunit types, and their relationship to other AChBPs. We collected more structural and functional information by reducing and non-reducing SDS-PAGE, deglycosylation, and amorphous CaCO_3_ binding of *Bg*AChBP. We expressed both subunits in *E. coli* and monitored their reassembly products in the electron microscope. Thereby we found that *Bg*AChBP1 alone is capable of forming rosette-like particles.

We also discovered that the rosette protein is a regular pentagonal dodecahedron. This quaternary structure is unique among the members of the Cys-loop receptor superfamily which otherwise occur only as pentamers or occasionally di-pentamers. Therefore, the major goal of the present study was to visualize the dodecahedron three-dimensionally in molecular detail to unravel specific features that enable *Bg*AChBP to form this unusual quaternary structure. At the subunit level, we conducted homology modeling. To visualize the quaternary structure, we performed three-dimensional electron cryo-microscopy (3D–EM) of the native rosette-like particle, and docking of the homology models to the resulting 6-Å cryo-EM structure. This yielded atomistic models of the entire dodecahedron which reveal the molecular architecture and indicate the inter-pentamer contacts.

## Results

### Biochemical and Sequence Analysis of *Bg*AChBP

**Figure 1:**
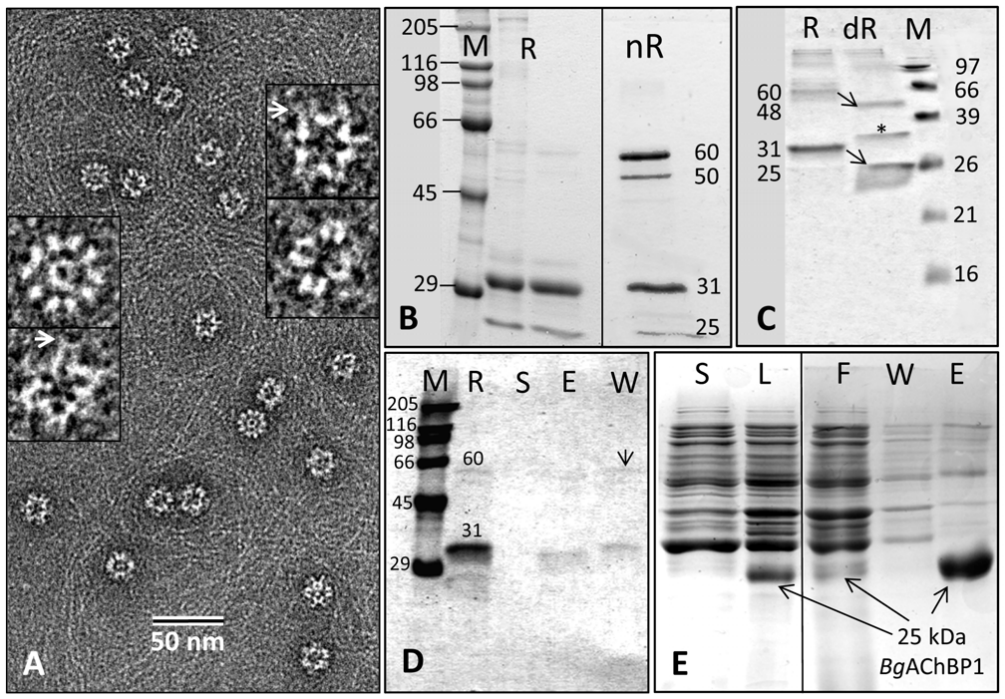
Electron microscopy and SDS-PAGE of *Bg*AChBP. **(A)** Negative staining EM of chromatographically purified rosette protein. 2% uranyl acetate was applied. Enlarged examples are also shown; note the peripheral protrusions (arrows). **(B)** Chromatographically enriched *Bg*AChBP (R, from “rosette protein”) under reducing (R) and non-reducing (nR) conditions. Note that in the second case, some material migrates as subunit dimers, whereas other material remains in the monomeric state. M, marker proteins. **(C)** *Bg*AChBP in glycosylated (R) and deglycosylated (dR) form, indicating that the 31 and 60 kDa bands represent the glycosylated and the 25 and 50 kDa bands the deglycosylated form (arrows). Asterisk, N-glycosidase F as deduced from controls; M, marker proteins. **(D)** *Bg*AChBP material extracted through binding to amorphous CaCO_3_. R, *Bg*AChBP starting material; S, supernatant after extraction; E, fraction eluted from CaCO_3_ by EDTA; W, wash buffer prior to EDTA extraction; M, marker proteins. Note that the wash buffer contains subunit dimers (arrow) and the slower migrating portion of the 31 kDa band, whereas the eluent contains its faster migrating portion. **(E)** Recombinant expression of *Bg*AChBP1. S, bacterial cell supernatant; L, bacterial cell lysate; F, flow-through of Ni column; W, wash buffer of Ni column; E, eluent of Ni column, rich in recombinant *Bg*AChBP1.

**Figure 2:**
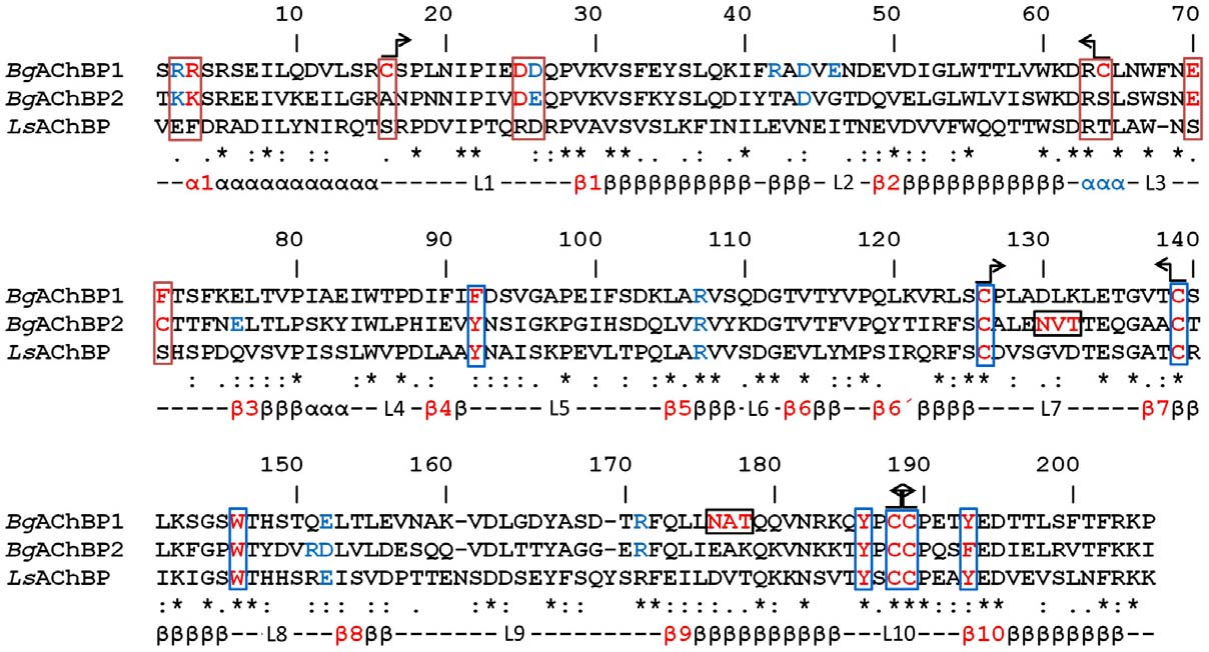
Sequence alignment of the *Bg*AChBP subunits and *Ls*AChBP (from *Lymnaea stagnalis*). The red residues are addressed in the main text in the context of ligand binding (blue boxes), inter-pentamer linkage (red boxes), N-glycan binding (black boxes), or disulfide bridges (arrow symbols). The blue residues probably form salt bridges between adjacent subunits within the same pentamer (see Fig. 4E). Note the specific exchanges Y92→F92 in *Bg*AChBP1 and Y193→F193 in *Bg*AChBP2. Also note the strictly conserved disulfide bridges stabilizing the eponymous Cys-loop L7 and the gating C-loop L10, the putative additional disulfide bridge C16↔C64 in *Bg*AChBP1, and the single cysteine C71 in *Bg*AChBP2. (Chain-specific residue numbers are given.) The secondary structure elements predicted from the published crystal structures are also indicated (L, loop). The short helix following strand β2 and marked in blue is absent in the molecular models of the BgAChBP subunits. Genbank entries JQ814367, JQ814368, AAK64377.

**Figure 3:**
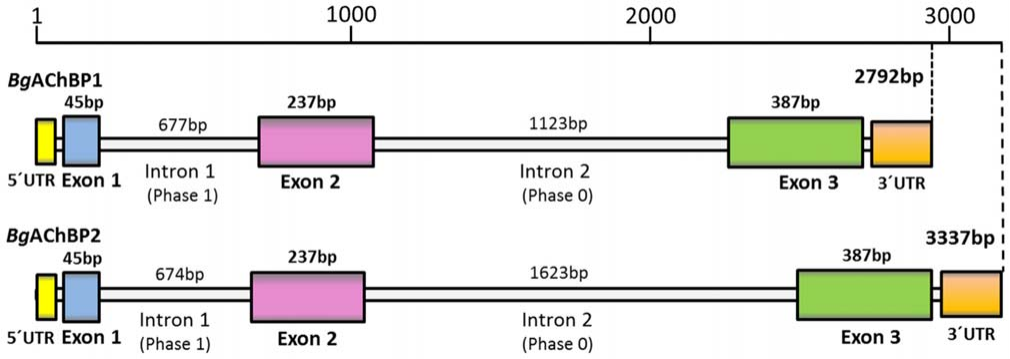
Gene structure of *Bg*AChBP1 and *Bg*AChBP2. Data retrieved from the preliminary *B. glabrata* genomic data (http://129.24.144.93/blast_bg/2index.html). Exon 1 and the first three amino acids encoded by exon 2 belong to the signal peptide, as deduced from evaluation in SignalP, and N-terminal protein sequencing (see Table 1). Genbank entries JQ814367, JQ814368.

The rosette protein (*Bg*AChBP) was highly enriched from *B. glabrata* hemolymph by ion exchange chromatography as published [16], which removed the large excess of multimeric hemoglobin present as deduced from electron microscopy (Fig. 1A). SDS-PAGE showed a strong band at 31 kDa and a minor band at 25 kDa (Fig. 1B), which agrees with our previous findings [16]. The 31 kDa band was excised, digested with trypsin, and generated fragments analyzed by mass spectrometry (Table 1). The obtained sequences of 17 tryptic peptides, and an N-terminal sequence obtained previously, were used for screening the EST and genomic library of the *B. glabrata* genome project (see Methods). We retrieved full-length sequences of two distinct subunits (termed *Bg*AChBP1 and *Bg*AChBP2), encompassing 205 amino acids plus signal peptide and sharing 52% sequence identity. The peptide sequences obtained by mass spectrometry covered 77% and 40% of the *Bg*AChBP1 and *Bg*AChBP2 sequences, respectively (see Table 1). A protein Blast revealed significant sequence identities (30–36%) to AChBP from the pond snail *Lymnaea stagnalis* (*Ls*AChBP), the planorbid snail *Bulinus truncatus* (*Bt*AChBP) and the sea hare *Aplysia californica* (*Ac*AChBP). A multiple sequence alignment with *Ls*AChBP is shown in Fig. 2; a corresponding identity matrix that includes additional related proteins is found in Table 2. Both primary structures were confirmed by sequencing cDNA clones obtained from *B. glabrata* tissues, using appropriate primers. Moreover, the exon/intron structure of both polypeptides could be retrieved from the *B. glabrata* genome project database (Fig. 3).

**Table 1:** Fragments of *Bg*AChBP1 and *Bg*AChBP2 as detected in rosette protein material.

*Bg*AChBP1	*Bg*AChBP2
(158 amino acids out of 205 = 77%)	(83 amino acids out of 205 = 40%)
SRSEILQDVLSR (12)	
CSPLNIPIEDDQPVK (15)	PNNIPIVDEQPVK (13)
VSFEYSLQR (9)	SNECTTFNELTLPSK (15)
ADVENDEVDIGLWTTLVW (18)	VTFV (4)
CLNWFNEFTSFK 12	FGPWTYDVR (9)
ELTVPIAEIWTPDIFLFDSVGAPEIFSDK (29)	QVDLTTYAGGER (12)
VSQDGTVTYVPQLK (14)	PCCPQSFEDIELR (13)
LSCPLADLK (9)	
LETGVTCSLK (10)	*XKKSREEIVKEILGRAN (16)
VDLGDYASDTR (11)	
QYPCCPETYEDATLSFTFR (19)	

With one exception (asterisk), the fragments were obtained by mass spectrometry (nanoUPLC-ESI Q-TOF) after tryptic digestion of a 31 kDa band cut out from an SDS-PAGE gel. The marked sequence was obtained six years earlier by N-terminal protein sequencing of the 31 kDa band. For localization of the peptides, see Fig 2.

The theoretical isoelectric points and molecular masses of *Bg*AChBP1 and *Bg*AChBP2 as predicted from the amino acid sequence are 4.78/23,456 Da and 5.52/23,508 Da, respectively, corresponding in mass to the minor component (25 kDa) observed on SDS gels. By N-glycosidase F digestion, we could quantitatively transform the 31 kDa band into the 25 kDa band. This demonstrates that the protein subunits are highly glycosylated, and that the initially detected 25 kDa trace protein represents a deglycosylated fraction (Fig. 1C). Under non-reducing conditions, ∼50% of the *Bg*AChBP protein material migrated in the range of a 60 kDa major and a 50 kDa minor component, i.e. as subunit dimers (see Fig. 1B). In all probability, the dimeric material exclusively represents *Bg*AChBP2, as deduced from the unique presence of a single unpaired cysteine (C71) in its sequence (see Fig. 2).

The rosette protein is also present in the extrapallial fluid (not shown), and therefore available for shell growth processes. In preliminary binding studies with amorphous CaCO_3_, *Bg*AChBP was completely removed from the supernatant, but most of the *Bg*AChBP material was subsequently released from the CaCO_3_ pellet by the wash buffer (Fig. 1D). Some of this “low affinity” material migrated at 60 kDa in non-reducing SDS-PAGE, indicating that it is identical with *Bg*AChBP2 (see Fig. 1D). Another portion, migrating at 31 kDa, was subsequently released from the CaCO_3_ pellet by adding 0.5 M EDTA (see Fig. 1D). This “high affinity” material might therefore represent *Bg*AChBP1.

### Homology Modeling of *Bg*AChBP Subunits and Pentamers

Homology modeling of the *Bg*AChBP sequences as targets with published AChBP crystal structures from *L. stagnalis*, *B. truncatus* and *A. californicum* AChBP as templates was promising for several reasons: (i) The five proteins share 30–36% sequence identity (see Table 2); (ii) the crystal structures are very similar [1]–[3]; (iii) most of the residues that are strictly conserved in the pentameric Cys-loop receptor superfamily are also present in the two *Bg*AChBP polypeptides (see Fig. 2).

**Table 2:** Identity matrix of AChBP, ACCBP and nACHR-LBD proteins.

	*Bg*	*Bg*	*Ls*	*Bt*	*Ac*	*Hdh*	*Hdh*	*Pf*	*Ct*	*Tm*
	AChBP1	AChBP2	AChBP	AChBP	AChBP	ACCBP1	ACCBP2	ACCBP	AChBP	AChR-α
***Bg*** **AChBP1**	**100**	52	36	36	33	27	26	25	27	17
***Bg*** **AChBP2**	52	**100**	33	36	30	27	24	23	20	20
***Ls*** **AChBP**	36	33	**100**	44	34	23	25	23	18	20
***Bt*** **AChBP**	36	36	44	**100**	32	25	23	22	16	17
***Ac*** **AChBP**	33	30	34	32	**100**	27	31	26	25	21
***Hdh*** **ACCBP1**	27	27	23	25	27	**100**	43	30	22	27
***Hdh*** **ACCBP2**	26	24	25	23	31	43	**100**	27	24	25
***Pf*** **ACCBP**	25	23	23	22	26	30	27	**100**	25	26
***Ct*** **AChBP**	27	20	18	16	25	22	24	25	**100**	27
***Tm*** **AChR-α**	17	20	20	17	21	27	25	26	27	**100**

Values were calculated using the CLUSTAL W multiple sequence alignment underlying the phylogenetic tree shown in Fig. 12.

**Figure 4:**
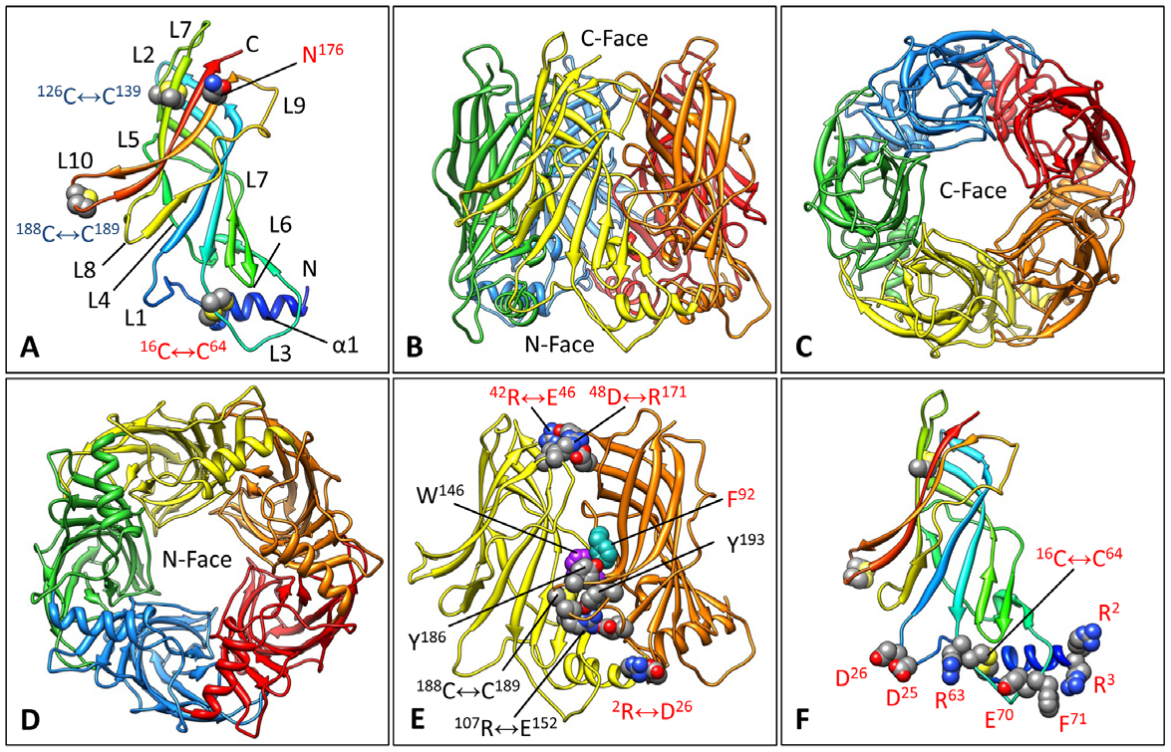
Homology models of *Bg*AChBP1. **(A)** The modeled subunit showing the N-terminal helix α1, the 10-stranded β-sandwich, the connecting loops L1 to L10, the three disulfide bridges, and the potential attachment site for N-linked glycans. **(B–D)** The modeled pentamer in side view (B) and the two different top views (C, D). The C-face is defined by the five C-termini and eponymous Cys-loops L7, the N-face contains the five N-termini and α1 helices. **(E)** Two neighboring subunits extracted from the modeled pentamer, with amino acid residues in the principal side of the ligand-binding pocket highlighted. Note that instead of phenylalanine F92, other AChP-LBD/AChBP members possess a tyrosine. Putative salt bridges connecting both subunits are also shown. **(F)** The modeled subunit showing the three disulfide bridges and the amino acids presumably involved in inter-pentamer contacts. Red labels mark features that are specific for *Bg*AChBP. (PDB-ID of the BbAChBP1 pentamer: 4AOD; PDB-ID of the *Bg*AChBP2 pentamer: 4AOE).

Homology models of *Bg*AChBP1 and *Bg*AChBP2, each encompassing 205 amino acids, were calculated as described in the Methods. Crystal structures of nAChR-LBD and AChBP subunits exhibit a 10-stranded β-sandwich core, an N-terminal α-helix, and the connecting loops L1 to L10 [1]-[3]. These features are also present in our homology models as shown here for subunit *Bg*AChBP1 (Fig. 4A). Functionally important loops in nAChR-LBD/AChBP are, for example, L10 (double-cysteine C-loop), L7 (Cys-loop), and L3 (MIR loop). In nAChR the MIR loop is exposed and bears, in human muscle nAChR, an epitope in the autoimmune disease myasthenia gravis [22].

Both *Bg*AChBP subunits possess the two disulfide bridges that are strictly conserved in this protein superfamily, C126↔C139 in loop L7 and C188↔C189 in loop L10. In addition, *Bg*AChBP1 shows an unprecedented opportunity for a third disulfide bridge, C16↔C64, connecting loops L1 and L3 (see Fig. 4A). This bridge is lacking in all other studied members of the Cys-loop receptor superfamily, and it is also absent in *Bg*AChBP2 (see Fig. 2). The potential attachment site in *Bg*AChBP1 for N-linked glycans, N176, is localized next to the C-terminus (see Fig. 4A). The corresponding site in *Bg*AChBP2, N130, occurs in the neighboring Cys-loop L7 (see Fig. 2).

From five copies of the homology-modeled subunits we built typical nAChR-LBD/AChBP pentamers (Fig. 4B–D), using the crystal structure of an *Ac*AChBP pentamer [23] as template. If viewed along its five-fold symmetry axis, such a pentamer shows two different faces: the “C-face” (containing the five C-termini; the basal face in AChR-LBD), and the “N-face” (containing the five N-termini; the apical face in AChR-LBD). According to the deglycosylation experiment documented in Fig. 1C, the AChBP subunits are glycoproteins. The position of the potential attachment site for N-linked glycans in the vicinity of the C-terminus (see Fig. 4A) suggests that the carbohydrates protrude from the C-face of the pentamer, rather than from the N-face, as in many other members of the nAChR-LBD/AChBP family.

The five active sites of nAChR-LBD/AChBP pentamers are located between two adjacent subunits, as shown in Fig. 4E. The connection between such neighbors is reinforced by the conserved salt bridge R107↔E152/D152. *Bg*AChBP1 shows opportunities for a second inter-subunit salt bridge, namely R2↔D26 (see Fig. 4E); in *Bg*AChBP2, K2↔D26 might play the same role (see Fig. 2). Additional specific opportunities are D48↔R171 in both subunits, R42↔E46 in *Bg*AChBP1 (see Fig. 4E) and E76↔R151 in BgAChBP2 (see Fig. 2). In nAChR-LBD/AChBP pentamers, the ligand-binding pocket is gated by the double-cysteine loop L10. The pocket shows a conserved principal side (in Fig. 4E delivered from the orange subunit) and a more variable complementary side (in Fig. 4E delivered from the yellow subunit). Examples for highly conserved residues in the pocket are Y92, W146, Y186, C188, C189 and Y193. In *Bg*AChBP1, Y92 is substituted for F92 (see Fig. 4E), and in *Bg*AChBP2, Y193 is substituted for F193 (see Fig. 2).

At the N-face, both *Bg*AChBP subunits exhibit charged and hydrophobic amino acids that are not conserved in other AChBPs. They are assumed to support the assembly of pentamers into higher-ordered structures as discussed below. The situation in *Bg*AChBP1 is shown in Fig. 4F. In *Bg*AChBP2, 2RR3 is substituted for 2KK3, and F71 is substituted for the single cysteine C71 (see Fig. 2).

### Recombinant *Bg*AChBP1 and BgAChBP2

**Figure 5:**
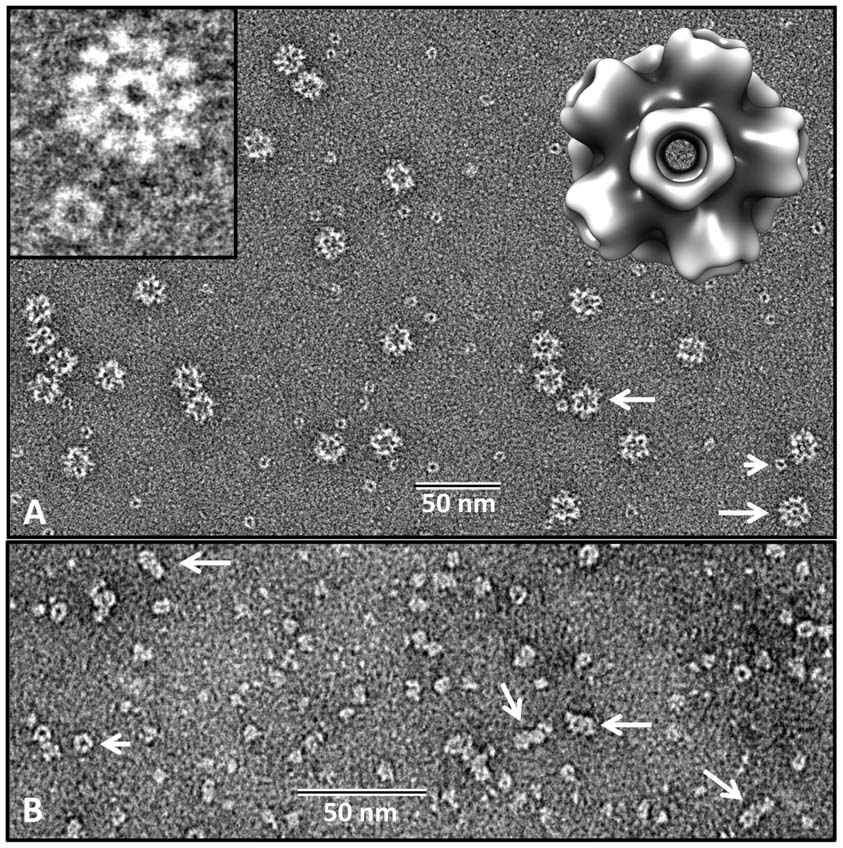
Electron microscopy of recombinant *Bg*AChBP1 and BgAChBP2 as expressed in *E. coli*. **(A)** Recombinant BgAChBP1 pentamers (short arrow) and dodecahedra (large arrows). Left insert, enlarged view along the five-fold symmetry axis of a recombinant dodecahedron and a single pentamer, respectively. Right insert, 3D reconstruction (resolution ∼20 Å) from ∼3000 negatively stained particles of the recombinant *Bg*AChBP1 dodecahedron. **(B)** Recombinant BgAChBP2 pentamers (short arrow) and presumed di-pentamers (large arrows). In several independent expression experiments, not a single dodecahedron was detected in the electron microscope.

Both *Bg*AChBP subunits were individually expressed in *E. coli*, solubilized from inclusion bodies, purified on a nickel column *via* a C-terminal His-tag (Fig. 1E), renatured, and studied in the electron microscope. Recombinant *Bg*AChBP1 formed small particles that we interpreted as pentamers, and much larger particles that are indistinguishable from the native rosette-like protein (Fig. 5A). From negatively stained images we produced a preliminary 3D reconstruction that revealed a regular pentagonal dodecahedron (right insert in Fig. 5A; ∼20Å resolution).

Recombinant *Bg*AChBP2 formed many pentamers, indicating that the protein refolding was successful, while rosette-like particles were completely absent (Fig. 5B). We frequently observed structures that might represent di-pentamers (arrows in Fig. 5B). However, in the electron microscope they are not clearly discernible from single pentamers lying together randomly, and our attempt to isolate a di-pentamer fraction was unsuccessful as yet.

### Three-dimensional Electron cryo-Microscopy (3D-EM) of *Bg*AChBP

**Figure 6:**
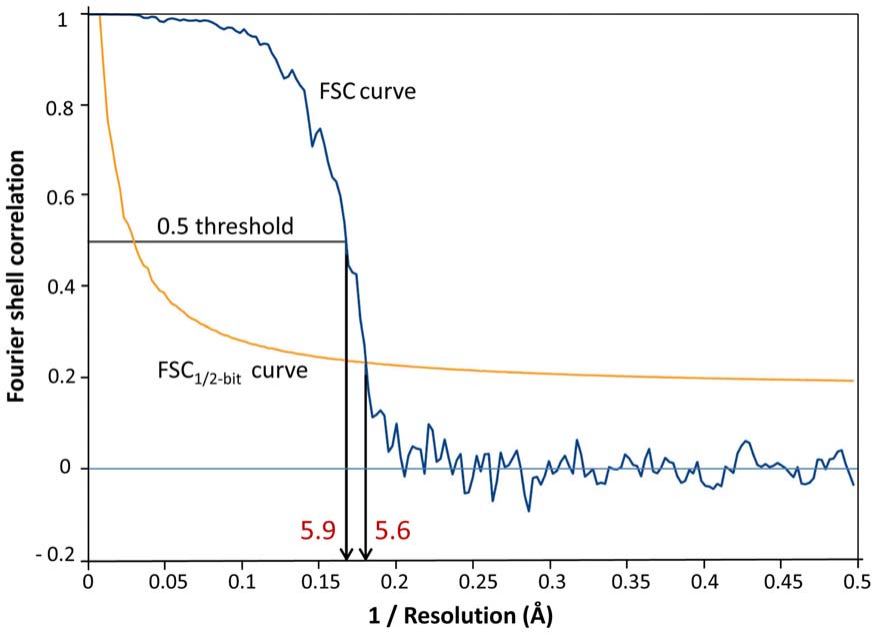
Resolution determination of the final 3D reconstruction of the *Bg*AChBP dodecahedron. The results for the density map in Fig. 8A–D are shown. Compared to the 5.9 Å obtained by the FSC_0.5_ criterion, the 5.6 Å determined with the FSC_1/2-bit_ criterion might be too optimistic. Therefore, this density map is further referred to as the “6-Å cryo-EM structure”.

**Figure 7:**
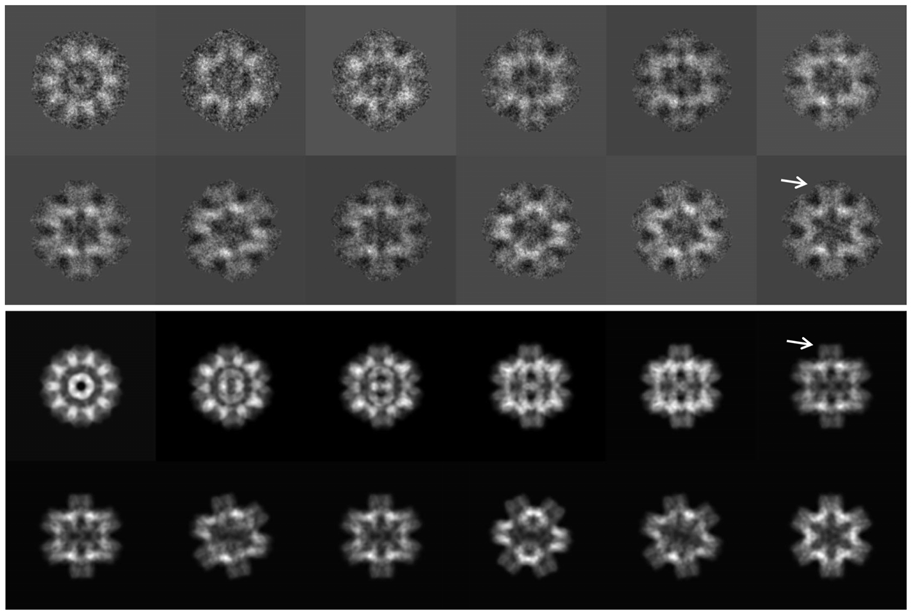
3D-EM processing of BgAChBP. Characteristic class sum images (top), and the corresponding reprojections (bottom) of the density map shown in Fig. 8A–D. Note peripheral protrusions in class sum images (upper arrow) that are absent in the corresponding reprojections (lower arrow). This disappearance results from masking for avoiding noise bias (see Methods).

**Figure 8:**
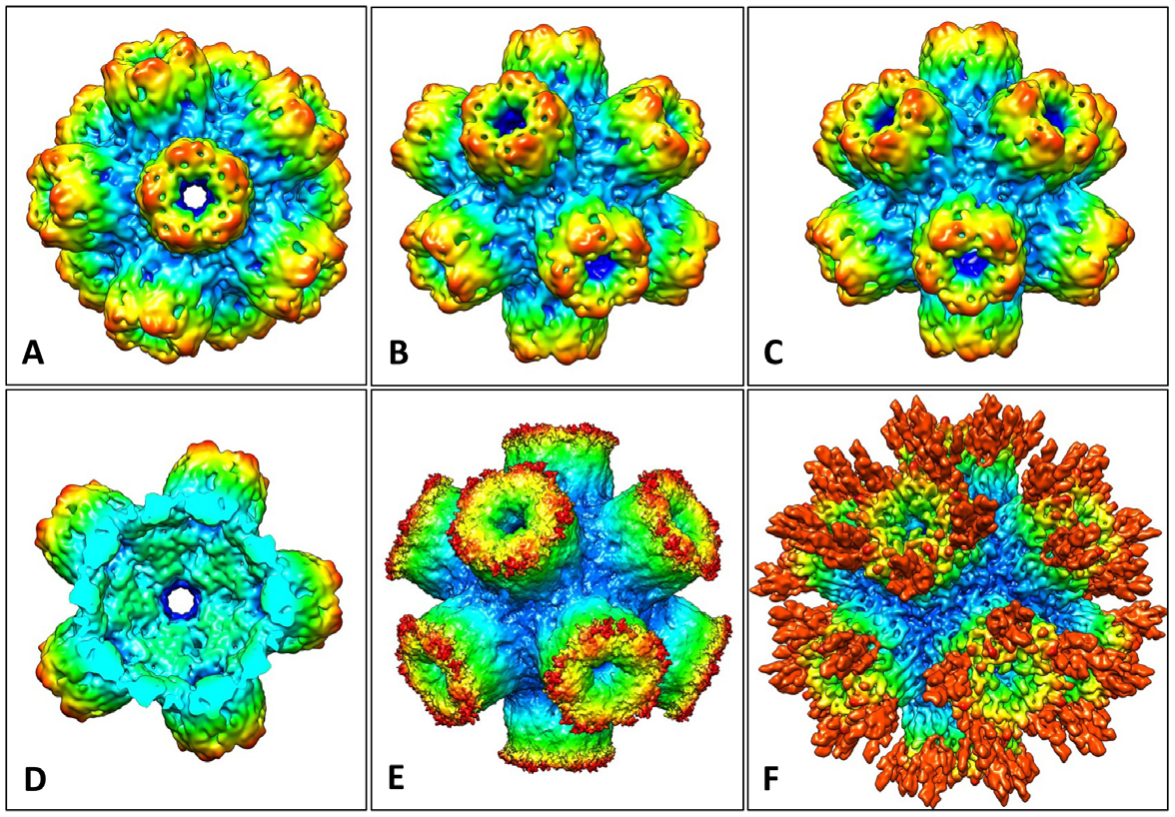
Cryo-EM structures of the *Bg*AChBP dodecahedron. **(A)** The final 6-Å cryo-EM structure viewed along one of six five-fold symmetry axes, exposing a central channel 2 nm in width. The overall diameter of the particle is 22 nm. **(B)** View along one of the 15 two-fold symmetry axes, exposing one of the 30 edges between two adjacent pentamers. (C) View along one of the 10 three-fold symmetry axes, exposing one of the 20 vertices at the junction between three neighboring pentamers. **(D)** Cut-open view to expose the central cavity (with the cut perpendicular to one of the five-fold axes of symmetry). **(E)** Unsharpened, unfiltered, unmasked version of the 6-Å cryo-EM structure to show the peripheral “fuzz” interpreted as glycans. **(F)** A 5.8-Å cryo-EM structure independently obtained from the same dataset. In this case, over-fitting of noise was accepted to avoid the loss of the putative carbohydrate side chains. (EM-DB ID of the 6-Å cryo-EM structure of the *Bg*AChBP dodecahedron: EMD-2055).

The native rosette-like particles were analyzed by 3D–EM as described for other proteins [24], [25]. We reached a resolution of ∼6 Å according to the 0.5 Fourier shell correlation (FSC_0.5_) criterion (Fig. 6). Representative class sum images and reprojections are shown in Fig. 7. The resulting cryo-EM structure is a regular pentagonal dodecahedron ∼22 nm in diameter and assembled from twelve ring-like pentamers; each pentamer measures 7–8 nm in height and width and comprises a central channel that is ∼2 nm in diameter (Fig. 8A). The 15 two-fold symmetry axes of the dodecahedron run through the 30 edges at which pentamers are in pairwise contact *via* a bridging mass (Fig. 8B). The 10 three-fold symmetry axes of the dodecahedron run through the 20 vertices (“triplexes”) at which three pentamers are joined by a central mass (Fig. 8C). The twelve central channels define the six five-fold symmetry axes of the dodecahedron and lead to a central cavity of ∼10 nm in diameter (Fig. 8D).

**Figure 9:**
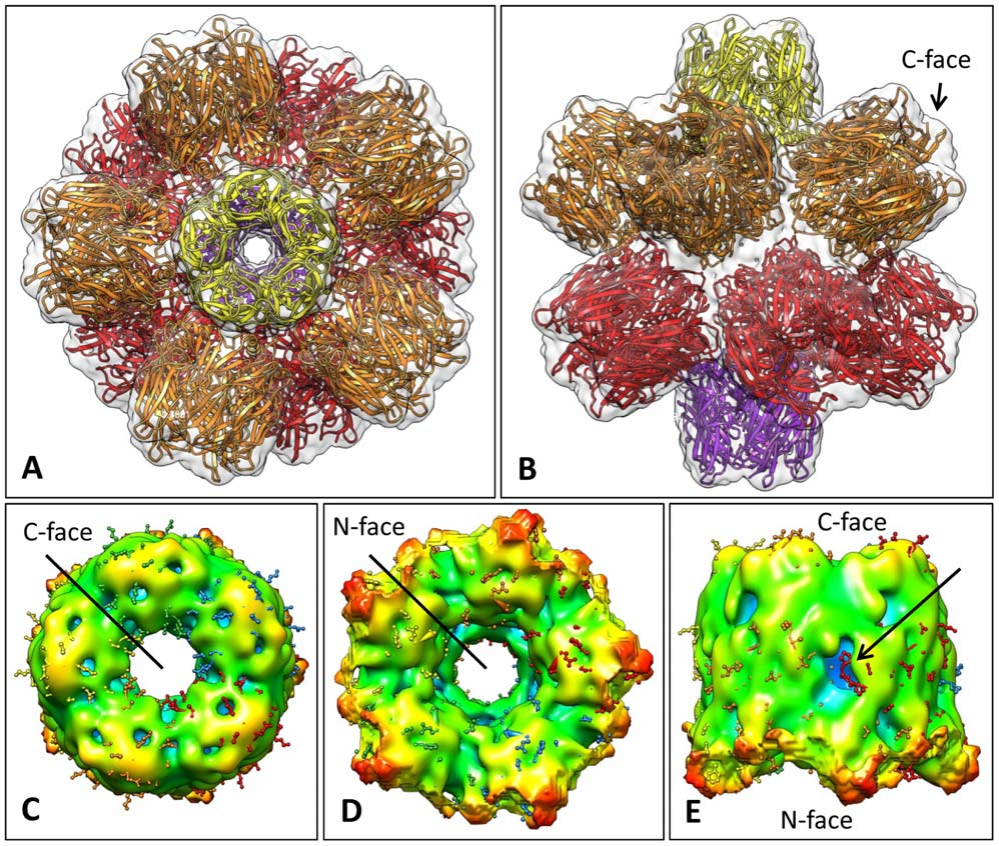
Docking of the molecular model of BgAChBP1 into the 6-Å cryo-EM structure. (A) The molecular model of the dodecahedron depicted along one of the five-fold symmetry axes. The 6-Å cryo-EM structure is shown in opaque to demonstrate the fitting. **(B)** The same model, viewed along one of the two-fold symmetry axes. **(C)** Top view of a pentamer extracted from the 6-Å cryo-EM structure, exposing the C-face; the docked molecular model is shown in ball & stick mode. **(D)** The same structure but rotated 180° to expose the N-face (the view from inside the central cavity). **(E)** The same structure in side view. Note the large cavity (blue) representing one of the five ligand-binding pockets, and the gating C-loop (arrow). Also note that in the cryo-EM structure the C-loop is too short to fully embed the molecular model (red) which might be due to its flexibility. (PDB-ID of the BbAChBP1 pentamer: 4AOD).

At an unfiltered, unsharpened pre-stage, the 6-Å cryo-EM structure has a fuzzy appearance at the exposed face of the pentamers (Fig. 8E). This indicates some flexible components in this region that were removed from the density map by filtering. An independent 3D-reconstruction from the same dataset, in which over-fitting of noise was not avoided, showed prominent protrusions instead of the fuzz (Fig. 8F). Although these protrusions might be dominated by noise bias, they support the idea of some flexible components in this region. Indeed, protrusions are directly visible in rosette protein particles (see arrows in Fig. 1A). They are also detected in class sum images, but absent in the corresponding reprojections (see arrows in Fig. 7); their disappearance is due to masking (see Methods). The exposed face of the pentamers turned out to be the C-face (Fig. 9). The localization of potential attachment sites for N-linked glycans at the C-face (see Fig. 4A) suggests that the flexible components are carbohydrate side chains. According to SDS-PAGE, the latter should encompass ∼20% of the molecular mass of the protein (see Fig. 1C).

### Docking of the Molecular Models into the 6-Å cryo-EM Structure

We docked twelve copies of the homology-modeled *Bg*AChBP1 pentamer (see Fig. 4B–D) into the 6-Å cryo-EM structure (Fig. 9A, B). Only slight readjustments occurred when we refitted the individual subunits as single rigid bodies. The correlation coefficient of the fitting was 0.89 as deduced from matching the cryo-EM structure and a density map that was simulated from the molecular model at 6 Å resolution. Comparable results were obtained with the *Bg*AChBP2 pentamer (not shown).

This docking clarified the handedness of the cryoEM structure, the specific rotation angle of the pentamers around their fivefold symmetry axis, and their overall orientation within the dodecahedron: They point with their C-face to the solvent (Fig. 9C) and with their N-face towards the central cavity (Fig. 9D). The docking enabled to identify the ligand-binding pocket and the gating C-loop in the cryo-EM structure (Fig. 9E). However, the C-loop of the molecular model was not fully embedded in the cryo-EM structure which has not enough mass in this region (see arrow in Fig. 9E). Underrepresentation of the C-loop in the filtered, sharpened density map indicates that this loop is rather flexible which agrees with its gating function at the entrance to the active site [23].

### The Putative inter-Pentamer Interface

**Figure 10:**
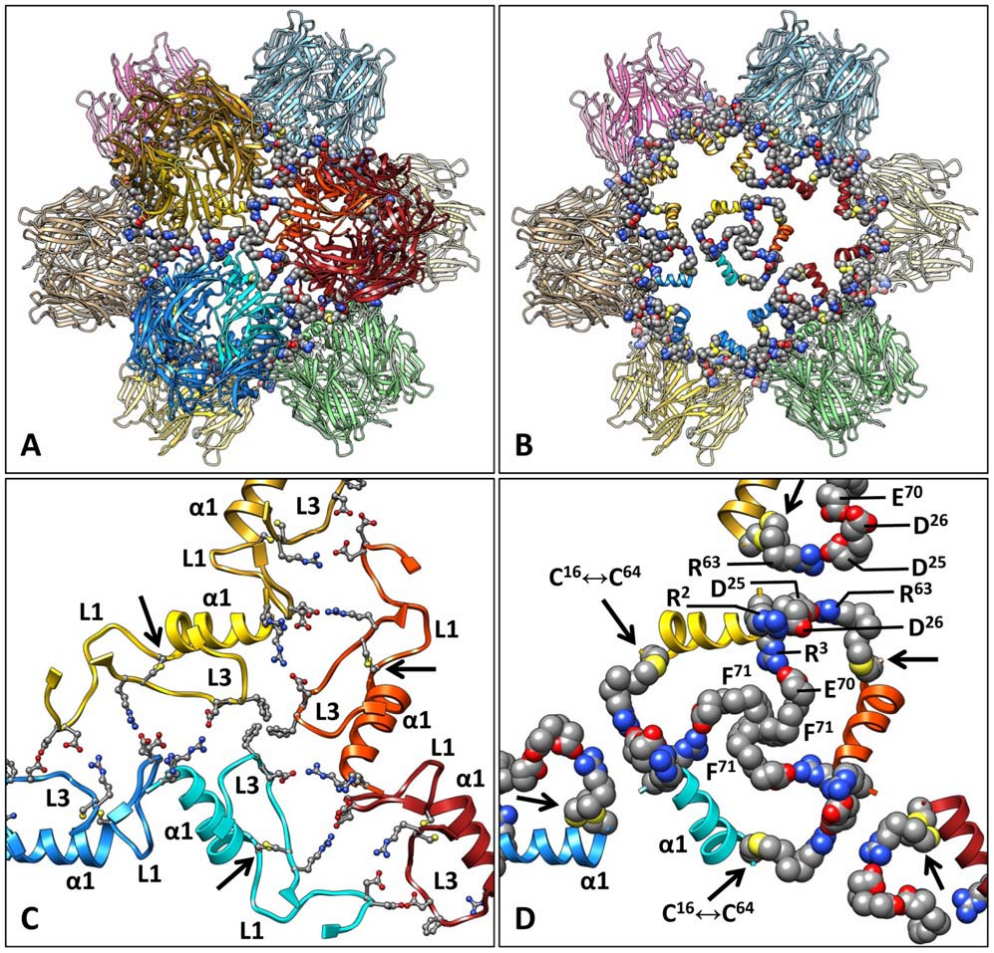
Putative inter-pentamer interfaces in a *Bg*AChBP1 dodecahedron. **(A)** Molecular model of the dodecahedron, viewed along one of the three-fold symmetry axes. The amino acid appositions providing opportunities for inter-pentamer bonding are highlighted. **(B)** The same view as in (A), with most of the three pentamers joined in the vertex removed. They are indicated by the yellow, light blue and orange red helices α1. In the center, the trigonal ring that makes the inter-pentamer contact is visible. **(C)** The residues that together with helix α1 form the trigonal ring, shown in ball & stick mode. In addition, loops L1 and L3 are indicated. Subunits of similar color stem from the same pentamer. Note the position of the C16↔C64 bridge (arrows). **(D)** Details of the central and three adjacent trigonal rings. Each ring connects three pentamers at their common vertex (*via* the F71 cluster, three salt bridges R3↔E70 and three salt bridges D25↔R63). Alternatively, two parallel salt bridges D25↔R63 can be considered as connection between two adjacent pentamers across their common edge. (PDB-ID of the BbAChBP1 pentamer: 4AOD).

Recombinant *Bg*AChBP1 is able to form a homo-oligomeric dodecahedron (see Fig. 5A), indicating that such a structure also exists within the animal. The 20 vertices of this model provide opportunities for chemical bonding between three adjacent pentamers (Fig. 10A). Each vertex contains a central trigonal ring (Fig. 10B). This ring is established by components from six subunits that are localized pairwise in the three pentamers (Fig. 10C). These components are helix α1 and residues from the N-terminal peptide (R2, R3), loop L1 (C16, D25, D26), and the “MIR” loop L3 (R63, C64, E70, F71). The position of these loops within the subunit is shown in Figs. 2 and 4A.

The trigonal ring encompasses six different structural elements, each present as three copies (Fig. 10D): (i) Helix α1 close to the N-terminus of each subunit; (ii) disulfide bridge C16↔C64 that is specific for *Bg*AChBP1 and tethers the C-terminal end of helix α1 to loop L3 of the same subunit (see also Fig. 4A); (iii) salt bridge R2↔D26 attaching the N-terminal end of helix α1 to loop L1 of a neighbored subunit from the same pentamer (see also Fig. 4E). These specific reinforcements of the N-terminal region of the *Bg*AChBP1 subunit might be required for withstanding the stretching forces inflicted by the other three elements that form the inter-pentamer bridges: (iv) salt bridge D25↔R63 connecting loops L1 and L3 of two subunits from adjacent pentamers; (v) salt bridge R3↔E70 joining the N-terminal end of helix α1 and loop L3 from two subunits of adjacent pentamers; (vi) a central hydrophobic cluster of three F71 residues delivered from three different pentamers.

In *Bg*AChBP2, the putative disulfide bridge C16↔C64 is lacking, but residues D25, R63 and E70 are conserved (see Fig. 2). R2, R3, D26 are substituted for K2, K3, E26, but these isofunctional exchanges suggest the same role in inter-pentamer binding. Instead of phenylalanine F71, the single cysteine C71 is present in *Bg*AChBP2. Disulfide-bridged subunit dimers have been observed (see Fig. 1B). The only possibility for this covalent dimerization exists *via* C71↔C71, since the other four cysteines in *Bg*AChBP2 form the canonical disulfide bridges in loop L7 and loop L10 (see Fig. 2 and 4A).

**Figure 11:**
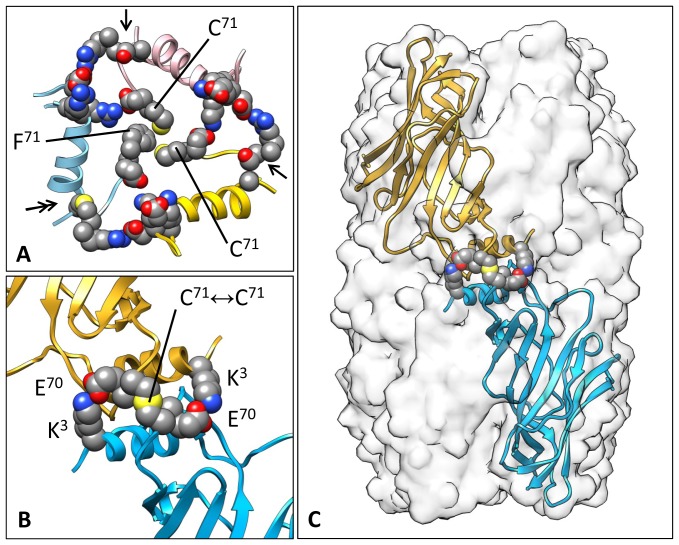
Putative inter-pentamer interfaces with *Bg*AChBP2. **(A)** Combination of a disulfide-bridged *Bg*AChBP2 subunit dimer and a *Bg*AChBP1 monomer in the vertex of a hypothetical hetero-dodecahedron. Note that in the trigonal ring, the covalent intra-subunit C16↔C64 bridge (double arrow) is lacking at two positions (arrows), because it is absent in *Bg*AChBP2 (that shows A16 and S64 instead). **(B)** Putative subunit dimer of *Bg*AChBP2 stabilized by an inter-subunit disulfide bridge C71↔C71 and two flanking salt bridges K3↔E70. The same subunit model as in (A) was applied. **(C)** Density map of a speculative *Bg*AChBP2 di-pentamer (opaque), simulated at 6 Å resolution from a molecular model of the di-pentamer. This model, of which one out of five subunit dimers is shown here, was constructed *in silico* by joining two *Bg*AChBP2 pentamers at their free C71 residues. (PDB-ID of the *Bg*AChBP2 pentamer: 4AOE).

*In silico*, we assembled a *Bg*AChBP2 homo-dodecahedron, with three C71 in the center of each vertex (not shown). However, such a particle might not exist *in vivo*, because we could not produce it from recombinant *Bg*AChBP2. Assuming that native *Bg*AChBP2 is present as subunit dimers with C71↔C71 connection, we see two other possibilities for oligo-pentameric assembly that both fit our observation that recombinant *Bg*AChBP2 did not form dodecahedra: (i) a hetero-dodecahedron that contains vertices combining a single *Bg*AChBP1 with a *Bg*AChBP2 dimer (Fig. 11A); (ii) a di-pentamer as indicated in Fig. 5B, with the pentamers linked by five disulfide bridges C71↔C71 and ten salt bridges K3↔E70 (Fig. 11B, C).

## Discussion

Recombinant *Bg*AChBP1 is capable of forming pentamers and dodecahedra (see Fig. 5A). Thus, subunit heterogeneity is not required for the latter. Inspection of the molecular model revealed special reinforcements of the pentamer (see Fig. 4A, E) and specific opportunities for inter-pentamer chemical bonding (see Fig. 10). Together, these variations explain how the dodecahedral architecture might be stabilized. In *Bg*AChBP2, at most of these positions either the same or an iso-functional amino acid is present. However, *Bg*AChBP2 lacks the putative internal disulfide bridge C16↔C64 that in *Bg*AChBP1 is considered as a major stabilizing element of the trigonal ring located in the vertices (see Fig. 10E and 11A). This fits the observation that recombinant *Bg*AChBP2 failed to form homo-oligomeric dodecahedra. Hetero-oligomers might be possible (see Fig. 11A), but experimental evidence for this is lacking. Alternatively, *Bg*AChBP2 might exclusively form di-pentamers, with C71↔C71 bridges as covalent linkers (see Fig. 11B, C). The presence of di-pentamers in recombinant *Bg*AChBP2 material (see Fig. 5B) and in the hemolymph has still to be confirmed. Nevertheless, the possible combination of *Bg*AChBP1 dodecahedra and *Bg*AChBP2 di-pentamers in *B. glabrata* is an additional stimulus for analyzing their differential expression, ligand-binding properties, and biological functions.

A regular dodecahedron is a Platonic solid with 60 rotational symmetries; it is the dual of an icosahedron which has the same symmetry. Such quaternary structures are well known from viruses. An example is the adenovirus type III dodecahedral penton particle that resembles the rosette protein in size and shape [26]. Dodecahedral quaternary structures are also known from some enzymes, for example lumazine synthase and erythrocyte peroxiredoxin-2 [27], [28]. Many molluscan hemolymph proteins are huge which reduces blood viscosity and colloid osmotic pressure [16], [24], [29]–[34], but are there more specific functional roles of the *Bg*AChBP dodecahedron? (i) It might enhance structural stability of the protein, thereby prolonging its existence and functionality in the hemolymph. (ii) The twelve assembled pentamers might interact allosterically during ligand binding. (iii) If *Bg*AChBP binds and neutralizes phytotoxins at picomolar affinities like other AChBPs [15], this might be improved by locally presenting 60 binding sites as in the dodecahedron. (iv) *Bg*AChBP binds amorphous CaCO_3_ and therefore might be involved in shell growth, like the ACCBPs [13], [14], [35]; for this, a highly ordered active site density as in the dodecahedron might be of relevance. (v) Genetically modified *Ls*AChBP pentamers were able to dock (with their C-face) to the membrane-bound pore domain of a serotonin receptor; these hybrid channels were then activated by acetylcholine [36]. One could therefore hypothesize that the *Bg*AChBP dodecahedron is capable of docking to and activating specific ion channels. This might even occur simultaneously at two opposing pentamers of the same dodecahedron, thereby bridging the gap between adjacent cell membranes (for which the 22 nm size of the protein would fit).

**Figure 12:**
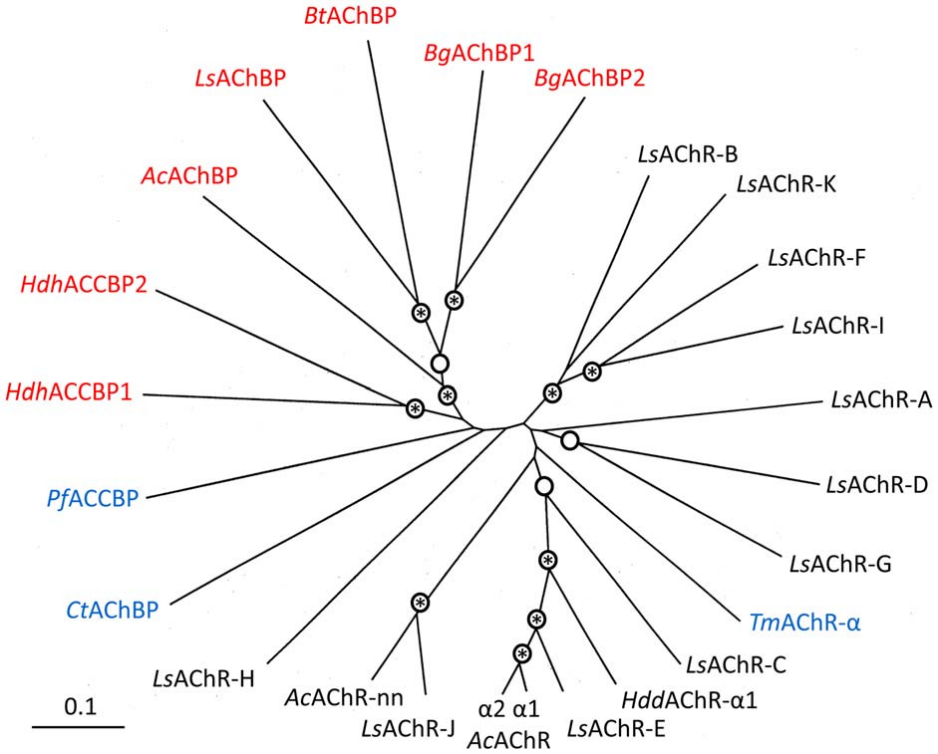
Radial phylogenetic tree of AChBP, ACCBP and AChR-LBD. Sequences of gastropod AChBP and ACCBP polypeptides (marked in red) are compared here to sequences of gastropod AChR-LBD polypeptides (marked in black). Sequences from the pearl oyster *P. fucata*, the polychaete annelid *C. telata* and the electric ray *T. marmorata* are also included (marked in blue). Nodes bootstrap-supported above 900 are indicated by a circle, those above 990 are additionally marked by an asterisk (1000 replicas were calculated). Note that the gastropod AChBP complex is clearly separated from the gastropod nAChR-LBD complex. Also note that *Bg*AChBP1 and *Bg*AChBP2 show a clear sister-group relationship, suggesting that they arose from a gene duplication event that occurred within the Planorbidae. The neighbor-joining method implemented in Clustal W was applied. A corresponding identity matrix is shown in Table 2. *Ac*, *Aplysia californica* (genbank entries AAL37250, AAL37251, AAL78648, AAL78649); *Bg*, *Biomphalaria glabrata* (JQ814367, JQ814368); *Bt*, *Bulinus truncatus* (PDB-ID 2BJ0); *Ct*, *Capitella teleta* (EY637248); *Hdd*, *Haliotis discus discus* (ABO26693); *Hdh*, *Haliotis discus hanei* (ABU51880, ABU62818); *Ls*, *Lymnaea stagnalis* (AAK64377, ABA60380 to ABA60390); *Pf*, *Pinctada fucata* (ABF13208); *Tm*, *Torpedo marmorata* (PDB-ID 2BG9); LBD, ligand binding domain.

The rosette protein represents only a low percentage of the hemolymph protein of *B. glabrata* which is greatly dominated by the multimeric hemoglobin [16], [37]. Thus, purification of the rosette protein in milligram amounts from these tiny animals is difficult. This has prevented not only solving the question of dodecahedron *versus* di-pentamer, but also a more detailed functional analysis. Therefore, the exact nature of the observed “low affinity” and “high affinity” CaCO_3_ binding *Bg*AChBP material remains uncertain. Nevertheless, specific CaCO_3_ binding was reproducible, and therefore at least a subfraction of the rosette protein (probably *Bg*AChBP1) could play a biological role in shell growth. In *H. discus hannai*, two homo-oligomeric acetylcholine-binding proteins have been detected. One appears to be a “true” pentameric AChBP involved in neuronal regulation, whereas the other one is a di-pentameric ACCBP [14]. Thus, apart from the oligomeric state, the situation resembles that in *B. glabrata*. However, a phylogenetic tree revealed that the subunit types of both animal species do not correspond; instead, they seem to result from two independent gene duplication events (Fig. 12).

This tree also shows that *Ls*AChBP and *Bt*AChBP form a common, strongly bootstrap-supported branch that does exclude *Bg*AChBP. This is remarkable, because *B. glabrata* and *B. truncatus* are members of the same family (Planorbidae), whereas *L. stagnalis* belongs to a related family (Lymnaeidae) of the same supertaxon Heterobranchia (which also includes *Aplysia* but excludes *Haliotis*). We assume that the more remote position of *Bg*AChBP in the tree reflects the changes in protein sequence, which *Bg*AChBP had undergone to become a dodecahedron. With respect to the phylogenetic origin of dodecahedral AChBP, it should be noted that all Planorbidae in which rosette-like proteins have been observed [16]–[21] belong to the same planorbid subtaxon that excludes *Bulinus* [38], [39]. To further trace the common root of these proteins, we included the available complete sequences of the ligand-binding domain of gastropod AChRs (from *L. stagnalis*, *A. californica* and *H. discus discus*) into our tree analysis. A clear result was that the AChBPs from the four heterobranch gastropods are monophyletic. Their specific relationship to abalone (*H. discus hannai*) and bivalve (*P. furcata*) ACCBP [13], [14], annelid (*Capitella teleta*) AChBP [4] and the various gastropod AChR types remains unclear, because the tree has not been convincingly solved at these splits. In contrast, it highly bootstrap-supports several interesting furcations of the AChR subtypes, for example a common branch of *Hdd*AChR-α1, *Ls*AChR-E and *Ac*AChRα1/α2 (see Fig. 12).

From vertebrates and insects, the exon-intron structure of various nAChR-LBD subtypes is available [40]–[43], but not from any mollusk. Gene structure information on AChBP/ACCBP is also lacking in the literature. In this context, the gene structure of the *Bg*AChBP subunits provided here (see Fig. 3) might be of particular interest in that it reveals an exon-intron pattern that is unprecedented, since only intron 1 resembles the situation in the non-molluscan species.

The icosahedral symmetry of *Bg*AChBP adds the powerful and rapidly improving 3D–EM method to the traditional repertoire of structure-function analyses of AChBP/AChR. The present resolution of ∼6 Å required less than 10,000 particles. Using recombinant dodecahedra and more particles, we expect to approach the 4 Å “magic goal”, as it has already been achieved for various viruses [44] and the membrane-bound domain of nAChR [6], amongst others. This would refine the inter-pentamer interface and unravel the functional state of the protein: Crystal structures of *Ac*AChBP complexed with ligand revealed that compared to apo-*Ac*AChBP, the C-loop shows an ∼7 Å smaller opening in complex with the agonists, and an ∼4 Å wider opening in complex with antagonists [23]. These positions of the C-loop correspond, in nAChR, to the three fundamental conformations of the ion channel: resting (closed, but capable of rapid activation), activated (open) and desensitized (closed) [6], [11], [23]. In this context, 3D–EM of dodecahedral *Bg*AChBP in complex with different ligands and inhibitors [45], [46] has the potential to further elucidate the structure of the resting, activated and desensitized states of AChBP/nAChR, with likely implications to the entire Cys-loop receptor superfamily.

## Materials and Methods

### Ethics Statement

The snail *Biomphalaria glabrata* stems from a long established culture in our institute. All animal work has been conducted according to relevant national and international guidelines.

### Biochemistry and nanoUPLC-MS/MS Analysis

Snail (*Biomphalaria glabrata*) maintenance and collection of hemolymph was done as previously described [16]. The animals were kept and bred in freshwater aquaria and fed daily on algae and chopped turnips. For hemolymph collection, the animals were numbed on ice, the foot punctured by a syringe needle, and the escaping hemolymph collected by a small pipette. Purification of rosette protein by anion exchange chromatography and SDS-PAGE (sodium dodecyl sulfate polyacrylamide gel electrophoresis) were performed as described [16]. The 31 kDa band was excised and subjected to reduction, alkylation, in-gel tryptic digestion, and subsequent mass spectrometric analysis (nanoUPLC-MS/MS  =  nanoscale ultra-performance liquid chromatography) as described previously [47]. Mass spectra were searched using Mascot v2.2 against an in-house compiled *B. glabrata* EST database supplemented with commonly observed contaminants (trypsin, human keratins). N-terminal sequencing of an excised 31 kDa band was performed by Dr. Hans Heid (DKFZ Heidelberg, Germany) in 2004 on a different batch. For protein deglycosylation, the N-glycosidase F deglycosylation kit from Roche (Mannheim, Germany) was applied. Protein binding to amorphous CaCO_3_ was assayed according to [13]. 1 mg of amorphous CaCO_3_ (courtesy of Prof. Wolfgang Tremel and Timo Schüler, JGU Mainz, Germany) was added to 200 µl *Bg*AChBP (5 mg/ml) and incubated for 3h at 4°C. The mixture was centrifuged for 10 min at 2000×g, the CaCO_3_ pellet washed 3x with 800 µl of 10 mM HEPES (4-(2-hydroxyethyl)-1-piperazineethanesulfonic acid), 0.5 M NaCl (pH 7.5), then dissolved in 0.5 M aqueous EDTA (ethylenediaminetetraacetic acid) and finally dialyzed against 10 mM Tris-HCl of pH 7.5 (Tris, tris(hydroxymethyl)aminomethane). Protein was monitored by SDS-PAGE.

### Databank Mining

Using the peptide sequences obtained from mass spectrometric analysis as queries, the *B. glabrata* EST's in Genbank were searched using tBlastn [48]. We obtained two EST entries (gi|163958251; gi|42722379) showing a significant similarity to AChBP from other gastropods and representing the N- and C-terminal part of a full-length polypeptide (termed *Bg*AChBP1) with the central fragment of *∼*15 amino acids lacking. A subsequent tBlastn search within the *B. glabrata* transcriptome (SRA, GenBank), using a concatenated sequence from both EST's, verified this chimeric sequence by overlapping coverage of 117 multiple reads. It also included the missing central peptide. Moreover, it recovered a second, related polypeptide (102 reads) that we termed *Bg*AChBP2. Together, both polypeptides cover the full range of tryptic peptides obtained by mass spectrometry (see Table 1). From preliminary *B. glabrata* genomic data (http://129.24.144.93/blast_bg/2index.html), the exon-intron structure of both AChBP genes was deduced by applying Genewise (http://www.ebi.ac.uk/Tools/Wise2/).

### cDNA Sequencing and Molecular Phylogeny

RNA was isolated from whole *B. glabrata* tissues using the automated Maxwell® 16 system (Promega), performed according to the manufacturer`s instructions. The RNA was stored at −20°C. Reverse transcription and amplification of the obtained cDNA was carried out using the SuperScript® III Reverse Transcriptase and Taq DNA polymerase (Invitrogen). Based on the retrieved sequences, gene specific primers were generated (primer sequences will be provided upon request), and both full-length primary structures could be verified by cDNA sequencing. Multiple sequence alignments were calculated with ClustalW2 [49], and the resulting phylogenetic trees visualized by TreeView [50]. Signal peptide prediction was done in Swissprot SignalP [51].

### Recombinant Protein Expression

Cloning of cDNA specific for both *Bg*AChBP types, and transformation of *E. coli* cells was done by standard techniques using the Gateway® system (Invitrogen). A His-tag was added at the respective C-terminus, pDEST™ 14-*Bg*AChBP prokaryotic expression vectors were constructed according to the manufactureŕs instructions, and BL21™ -AI cells were transfected. The recombinant protein was expressed in inclusion bodies that were separated by centrifugation (15,000g; 40 min), dispersed in 8M urea/1mM ß-mercaptoethanol, and then sonified. The recombinant protein was purified by affinity chromatography *via* Ni-NTA agarose (GE Healthcare), and then diluted for 18–24 h in “redox-shuffling refolding buffer” according to [14] (0.1 M Tris-HCl, 0.5 M L-arginine, 0.9 mM oxidized glutathione, 2 mM EDTA, pH 8.0) to a final concentration of *∼*30 µg/ml. The refolded protein was concentrated to *ca*. 3 mg/ml by Millipore ultrafilter units, dialyzed against 20 mM Tris-HCl buffer (pH 8.0), and subsequently concentrated to 30 mg/ml. Thereafter, the buffer was supplemented with imidazole (final concentration 50 mM) to prevent protein aggregation *via* the His-tag. Recombinant pentamers and dodecahedra were separated by gel filtration chromatography on Biogel A0.5m (Biorad) in 20mM Tris-HCl buffer, 50mM imidazole, pH 8.0. Protein fractions were analyzed by UV spectrometry, SDS-PAGE and electron microscopy.

### Electron Microscopy (EM)

Negatively stained samples were prepared as described [31] using 2% uranyl acetate, and imaged on an FEI Tecnai 12 TEM. Sample preparation for cryo-EM was conducted with a modified Gatan Cryoplunge™3. A protein concentration of 1.2 mg/ml was used on C-flat holey carbon grids (Protochips Inc., Raleigh, NC, USA) which were situated in an atmospheric chamber containing nitrogen gas and 95–97% humidity. The blotting time before dropping the sample into liquid ethane was 3 seconds. Images were taken with an underfocus range of 2–5 µm on an FEI Tecnai F20 TEM (Cs 2.0; voltage 200 kV; calibrated magnification 50,000×; nominal electron dose range 28–36 e-/Å^2^). They were recorded on Kodak SO-163 film**,** developed for 12 minutes at 21°C in Kodak D19 high contrast developer and fixed in Kodak AGEFIX fixer (Agfa-Gevaert, Mortsel, Belgium).

### Image Processing

The micrographs were digitized using a Heidelberg Primescan D7100 drum scanner with a step size of 5 µm, resulting in a resolution of 1 Å/pixel in the digitized images of the specimen. In order to prevent the formation of Newton rings, the micrographs were mounted onto the drum using a thin layer of oil (Mounting Fluid, SDS AG, Germany) between the drum and the micrographs. Effects of the contrast transfer function (CTF) were corrected by using the software findCTF2D [52]. The dataset (9770 single particles selected from 151 micrographs) was averaged to a resolution of 4 Å/pixel and low-pass-filtered to a resolution of 20 Å. A dodecahedral structure obtained using the multirefine algorithm of EMAN 1.9 [53] served as an initial model for refinement in EMAN 1.9. After calculating 13 iterations, gradually increasing the point-group symmetry from C1 to C5 and D5, the 3D reconstruction was subjected to icosahedral symmetry, yielding a structure which was in accordance with the last D5-symmetrical reconstruction calculated. Subsequently, the dataset was processed with icosahedral point-group symmetry in two different ways:

- Over-fitting of noise avoided: In a refinement cycle of 15 iterations, the full resolution dataset at 1 Å/pixel and 1° references were used. To prevent noise bias, the high frequency cut-off value of the filtered dataset was set to 8 Å for the alignments and to 3 Å for the reconstructions. Furthermore, the 3D reconstruction of each iterative cycle serving as a reference for the subsequent alignment was low-pass filtered to 10 Å. Amorphous protrusions, which from the start appeared at the exposed face of the pentamers, were carefully masked out after each iterative cycle in order to prevent noise bias in this region. The final density map was calculated using 8058 selected particles. A Fourier shell correlation curve calculated from two reconstructions (each containing one half of the dataset) indicated a resolution of 5.9 Å according to the 0.5-threshold. A negative temperature factor of 278.9 Å^2^ was applied using the computer program bfactor 1.04 (Nikolaus Grigorieff: http://emlab.rose2.brandeis.edu/bfactor). The resulting cryo-EM structure (see Fig. 8A–D) was low-pass filtered to 6 Å. (For the unsharpened, unfiltered and unmasked density map, see Fig. 8E.).
- Over-fitting of noise accepted (to study the protrusions as putative glycans): In a refinement cycle of 85 iterations, the high frequency cut-off values were modified according to the resolution measured; the last 25 iterations were calculated using the full resolution dataset at 1 Å/pixel. The amorphous protrusions were not masked out, although they should at least partially represent noise bias. The final density map (5.8 Å resolution) was calculated from 8347 selected particles. A negative temperature factor of 375.84 Å^2^ was applied using the software EM-BFACTOR [54], [55]. The resulting 3D reconstruction (see Fig. 8F) was low-pass filtered to 6 Å.

### Homology Modeling, Rigid-body Fitting, and Visualization

Homology modeling with *Ac*AChBP as template (PDB-ID 2BR7 [23]) and loop refinement were performed in Modeller 9v9 [56]; the results were controlled in MolProbity 3.18 [57]. Visualization, rigid-body fitting, rotamer refinements at interfaces and the molecular graphics were done in UCSF Chimera [58].
